# Searching for a change: The need for increased support for public health and research on fungal diseases

**DOI:** 10.1371/journal.pntd.0006479

**Published:** 2018-06-14

**Authors:** Marcio L. Rodrigues, Priscila C. Albuquerque

**Affiliations:** 1 Instituto Carlos Chagas (ICC), Oswaldo Cruz Foundation (Fiocruz), Curitiba, Brazil; 2 Instituto de Microbiologia Paulo de Góes, Federal University of Rio de Janeiro, Rio de Janeiro, Brazil; 3 Center for Technological Development in Health, Fiocruz, Rio de Janeiro, Brazil; University of Tennessee, UNITED STATES

## Introduction: The threat of fungal diseases

Fungal diseases have been continually neglected over the years despite their alarming impact on human health. Recent estimates [1] suggest a global annual occurrence of approximately 3,000,000 cases of chronic pulmonary aspergillosis, more than 200,000 cases of cryptococcal meningitis, 700,000 cases of invasive candidiasis, 500,000 cases of *Pneumocystis jirovecii* pneumonia, 250,000 cases of invasive aspergillosis, 100,000 cases of disseminated histoplasmosis, over 10,000,000 cases of fungal asthma, and 1,000,000 cases of fungal keratitis. It is estimated that fungal infections kill more than 1.5 million people every year [2]. Morbidity due to fungal infections is also significant. Chromoblastomycosis and eumycetoma, for instance, cause debilitating diseases affecting subcutaneous tissues, skin, and underlying bones [3,4]. Importantly, the epidemiology of fungal diseases is dynamic and hard to predict. Recently, the multidrug-resistant pathogen *Candida auris* has emerged as a serious threat to human health, with some infections resistant to all main classes of antifungal medications [5]. Known diseases also raise new concerns. The city of Rio de Janeiro, Brazil, currently faces the largest epidemic of sporotrichosis in history [6]. Paracoccidioidomycosis is still one of the most important systemic mycoses in Latin America and the leading fungal cause of mortality in non-immunosuppressed individuals in Brazil [3,7].

Mortality and morbidity due to fungal diseases mostly affect individuals living under socioeconomic restrictions [8,9]. Reliable and cost-effective diagnostic tools are available for a very limited number of mycoses, and therapeutic options are restricted to a few classes of poorly effective, toxic, and expensive pharmaceutical agents [10]. There are no licensed antifungal vaccines [12]. The last antifungals approved for clinical use were the echinocandins in 2002 [11]. According to the Global Fund for Fungal Infections (GAFFI), several key antifungals also are not available or not even registered in multiple regions where fungal diseases are most lethal [2]. These serious gaps in diagnostic, therapeutic, and preventive tools for fungal infections are a dangerous combination that results in high costs and low efficacy of clinical approaches [13]. To minimize the number of deaths caused by fungal diseases, novel therapeutic, diagnostic, and preventive tools must be developed, and the diagnostic tests and antifungal agents that are available must be rationally deployed.

## Actions to combat fungal diseases

**Fig 1:**
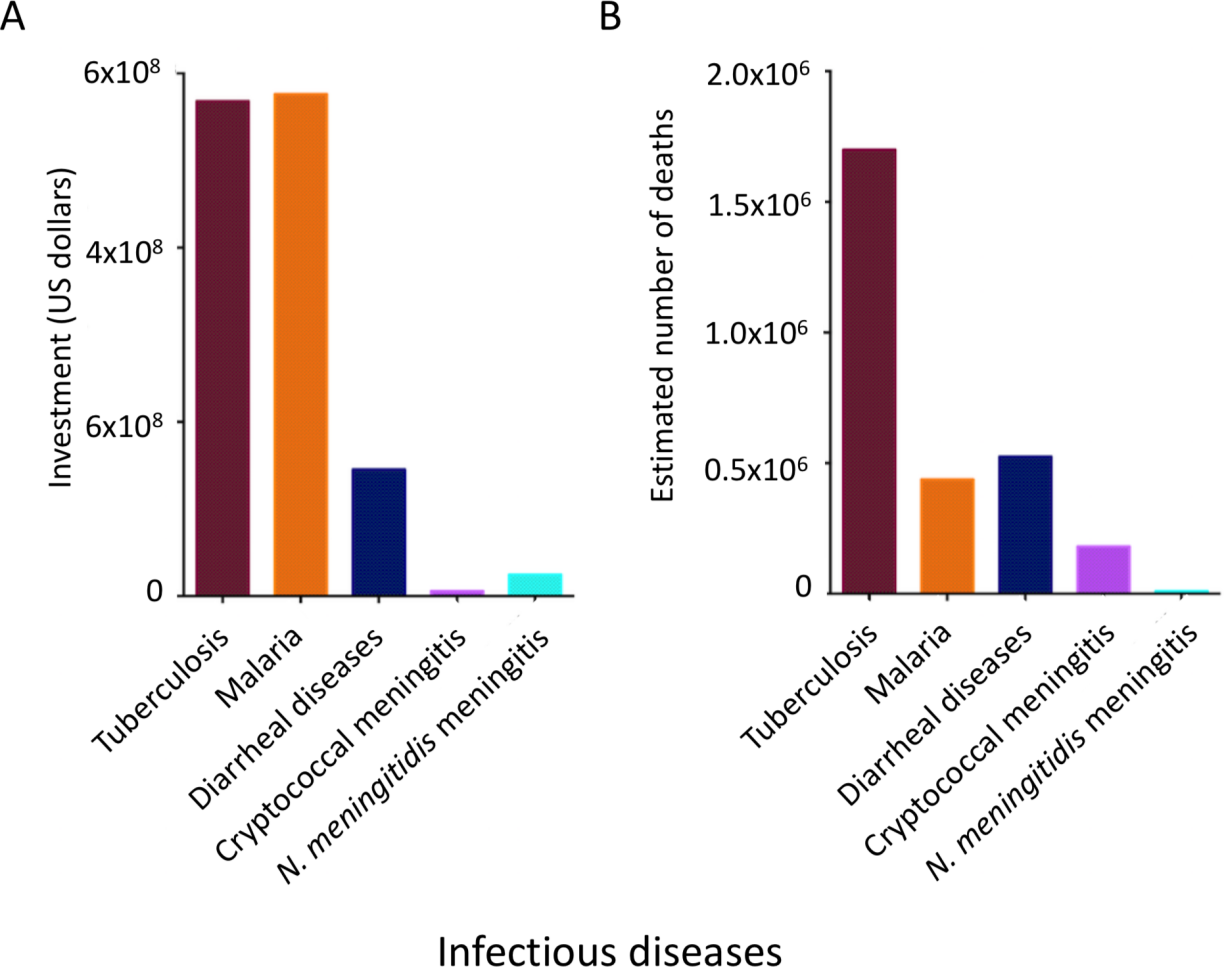
**Analysis of 2016 total investments (A) and annual estimation of human deaths (B) associated with tuberculosis, malaria, diarrheal diseases, and meningococcal or cryptococcal meningitis.** Investment/death ratios for each disease corresponded to approximate values of 2,458 (meningococcal meningitis), 1,315 (malaria), 334 (tuberculosis), 276 (diarrheal diseases), and 31 (cryptococcal meningitis).

The complex scenario posed by fungal diseases demands novel solutions, but concrete actions are not common. Although directed programs have been recently established for treating mycetoma [14], most mycoses still demand attention from public health authorities and decision makers. Funding for fungal diseases is incredibly small compared to funding available for infectious diseases causing similar mortality (Fig 1 and [15]). Mycetoma, chromoblastomycosis, and “other deep mycoses” have been recently included in the World Health Organization (WHO)’s list of neglected tropical diseases, but specific information on WHO’s plans to fight fungal diseases is still not publicly available [16]. Cryptococcal meningitis, which ranks amongst the most poorly funded diseases in the world, has recently been proposed as a neglected tropical disease [17].

## Funding for fungal diseases

The current scenario is incompatible with an optimistic perspective, as illustrated by the most recent edition of the Global Funding of Innovation for Neglected Diseases (G-Finder) Report [18]. Cryptococcal meningitis was the only fungal disease with measurable funding mentioned in the report. Other fungal diseases were not included in the report, denoting negligible funding. This is consistent with earlier estimates for research and development funding for human cryptococcosis that revealed indices of support that were greatly disproportionate to its importance for global health in comparison to other neglected diseases [15]. Cryptococcosis was classified within the most poorly funded neglected diseases covered by the G-Finder survey, receiving less than 0.5% of global funding [18]. According to statistics obtained from WHO’s public databases, cryptococcal meningitis is the fourth most deadly infectious disease (excluding HIV), after tuberculosis (approximately 1,700,000 deaths [19]), diarrheal diseases (approximately 525,000 deaths [20]), and malaria (approximately 438,000 deaths [21]). However, according to the 2017 G-Finder report [18], scientific activities in the field of cryptococcal meningitis received approximately 100-fold reduced funding in comparison to malaria and tuberculosis and 25-fold reduced funding in comparison to diarrheal diseases. For a more direct disease comparison, cryptococcal meningitis kills 20 times more humans than the brain disease caused by the bacterial pathogen *Neisseria meningitidis* [22]. Nevertheless, funding for research on bacterial meningitis was 4.35-fold higher than that available for the fungal disease (Fig 1 and [18]).

Other clinically relevant but neglected mycoses, including paracoccidioidomycosis, mycetoma, sporotrichosis, and chromoblastomycosis, were not even mentioned in the G-Finder report [18], suggesting that these areas of research received negligible funding. We used publication data (2017) from the Web of Science database (https://clarivate.com/products/web-ofscience/databases/) to probe the impact of this lack of funding. For comparison, we included tuberculosis and malaria, for which well-established programs of funding are available [18]. We found that tuberculosis and malaria were the focus of 8,827 and 5,687 articles published in 2017, respectively. In contrast, fungal diseases were much less investigated, producing 213 (cryptococcosis), 80 (paracoccidioidomycosis), 51 (chromoblastomycosis), 53 (mycetoma), and 56 (sporotrichosis) articles in the same period (Table 1).

**Table 1:** Analysis of publication records and reported funding in the fields of tuberculosis, malaria, cryptococcosis, paracoccidioidomycosis, mycetoma, sporotrichosis, and chromoblastomycosis^a^.

Disease^b^	Number of articles in 2017	Number of articles reporting funding organization^c^							N^d^	Percent of articles^e^
		NIH	Gates	NFSC	WT	EC	MRC	ERC		
CBM	51	0	0	7	0	0	0	0	7	13.7
Mycetoma	53	0	0	1	0	0	0	0	1	1.9
Sporotrichosis	56	1	0	2	0	0	1	0	4	7.1
PCM	80	6	0	0	0	0	0	0	6	7.5
Cryptococcosis	213	25	0	10	5	2	4	2	48	22.5
Malaria	5,687	658	340	132	325	134	133	43	1,765	31.0
Tuberculosis	8,827	920	180	452	196	305	147	45	2,245	25.4

^a^Publication records were retrieved from the Web of Science database (Clarivate). Articles containing each disease nomenclature in the title and abstract sections were selected for further analysis. Results were imported from the database as raw data files and processed with the data-mining software VantagePoint 9.0 (Search Technology Inc). Funding organizations were standardized using the VantagePoint cleanup tool, followed by manual proofreading to ensure that the funding agencies were accurately described.

^b^CBM, chromoblastomycosis; PCM, paracoccidioidomycosis.

^c^NIH, National Institutes of Health, United States of America; Gates, Bill and Melinda Gates Foundation, US; NSFC, National Natural Science Foundation of China; WT, Wellcome Trust, United Kingdom; EC, European Commission; MRC, Medical Research Council, UK; ERC, European Research Council.

^d^Number of articles reporting funding from at least one major organization.

^e^Percentage of articles reporting funding from at least one major organization.

We further examined the authors' statements of financial support for major international agencies with a history of support of research into neglected diseases [23], such as the National Institutes of Health, USA, the Bill and Melinda Gates Foundation, the National Natural Science Foundation of China, the Wellcome Trust, the Medical Research Council, the European Commission, and the European Research Council. We found that 22.5% to 31% of the articles published in the fields of cryptococcosis, malaria, and tuberculosis received financial support from at least one major funding organization, corresponding to 2,245 articles in the field of tuberculosis, 1,765 articles in the field of malaria, and only 48 articles in the area of cryptococcosis. In the chromoblastomycosis field, 13.7% of the studies reported major financial support, with the particularity that all these articles (*n* = 7) received funding from the National Natural Science Foundation of China. The general picture was more serious in the fields of paracoccidioidomycosis, sporotrichosis, and mycetoma. Funding from major organizations was reported in only six articles focused on paracoccidioidomycosis (7.5%) and four publications in the area of sporotrichosis (7.1%). Only one article reported support from major funding agencies in the field of mycetoma (1.9%). These results are all summarized in Table 1.

## Time for action

Health emergencies such as the recent Zika outbreak demand rapid responses [24], but responses are similarly necessary to combat diseases that have been devastating different nations for years. Recently, there have been numerous calls for change in the current scenario of research, development, innovation, and public health in the field of fungal diseases [3,9,15,17,25–27]. We recognize that changes of this magnitude are complex and hard to achieve, especially in times of crisis involving economic and public health issues [28]. For fungal diseases, the need for change is unquestionable: it is time for action.
